# The exercise and concussion health study (TECHS): Pilot and feasibility protocol

**DOI:** 10.1016/j.conctc.2026.101608

**Published:** 2026-01-29

**Authors:** Emma M. Tinney, Mark C. Nwakamma, Goretti España-Irla, Madeleine Perko, Ryan Luke Sodemann, Jacqueline Caefer, Julia Manczurowsky, Charles H. Hillman, Alexandra Stillman, Timothy P. Morris

**Affiliations:** a Department of Psychology, Northeastern University, Boston, MA, USA; b Institute for Cognitive & Brain Health, Northeastern University, Boston, MA, USA; c Department of Physical Therapy, Movement, & Rehabilitation Sciences, Northeastern University, Boston, MA, USA; d Department of Neurology, Beth Israel Deaconess Medical Center, Boston, MA, USA; e Department of Applied Psychology, Northeastern University, Boston, MA, USA

## Abstract

Mild traumatic brain injury (mTBI) is a prevalent neurological condition, affecting millions worldwide, and frequently resulting in persistent cognitive impairment that significantly impacts daily functioning. Current clinical management and recommendations lack evidence based therapeutic interventions, with treatment approaches limited to symptom monitoring and activity modification. Non-pharmaceutical interventions are needed to promote cognitive recovery and mitigate long-term consequences of mTBI. The goal of this randomized control pilot study is to evaluate the feasibility and preliminary efficacy of a symptom-guided and virtually delivered aerobic exercise intervention for improving cognitive function following mTBI. We are conducting a 12-week pilot randomized control trial of exercise to promote recovery from mTBI, which aims to recruit 24 participants who have suffered an mTBI within the last year and are between the ages of 18–55 years old. Participants are randomly assigned to one of two groups: an intervention group receiving 90 min of virtually-delivered, symptom-guided aerobic exercise weekly for 12 weeks, or a control group receiving 90 min of virtually-delivered balance exercises weekly for 12 weeks. Comprehensive assessments, including cognitive testing and multimodal neuroimaging, were conducted pre and post intervention. We implement physical activity monitoring during weeks 1, 6, and 12 using accelerometry to measure behavior changes. This study will establish the feasibility of virtual exercise delivery in mTBI populations and preliminary evidence regarding cognitive and brain health benefits of aerobic exercise in mTBI. Results will inform future large-scale trials and contribute to an accessible, evidence-based intervention for cognitive recovery after mTBI.

## 1 Introduction

Mild traumatic brain injury (mTBI) represents one of the most prevalent neurological conditions, affecting an estimated 60 million individuals worldwide annually [1]. mTBI is not limited to sport related injuries but affects the population at large, and not all individuals make a complete recovery [2,3]. Moreover, cognitive impairment specifically is a significant predictor of returning to work after injury [4], yet there is no FDA-approved treatment available to directly treat cognitive dysfunction after an mTBI, making the clinical management of mTBI particularly challenging [5]. Current clinical practice rely primarily on symptom management and activity modification, with limited evidence-based interventions available to promote active recovery and functional improvement [5]. The widespread nature of mTBI, combined with the significant percentage of patients experiencing persistent symptoms, creates an urgent public health need that current approaches fail to adequately address. This treatment gap highlights the need for accessible, cost-effective, and scalable non-pharmacological interventions that can be widely implemented to support the substantial population of individuals experiencing persistent post-concussive symptoms and cognitive deficits. Given these challenges in mTBI management, aerobic exercise emerges as a promising therapeutic modality that improves cognition and overall brain health in the general population [[6], [7], [8]], targeting the underlying neurobiological mechanisms (i.e., axonal damage, neuroinflammation) contributing to cognitive dysfunction following mTBI.

Multiple systematic reviews have summarized the effects of physical activity and aerobic exercise on TBI outcomes in humans [[9], [10], [11], [12], [13], [14], [15], [16]], but findings are inconsistent due to significant methodological heterogeneity, such as inadequate control groups, intervention timing, outcome measures, and study populations. These methodological shortcomings reveal the critical need for rigorously designed randomized controlled trials that employ standardized, evidence-based exercise protocols, with appropriate active control conditions, to establish definitive clinical guidelines for exercise prescription in mTBI recovery. Additionally, prior interventions were delivered in person [[17], [18], [19], [20], [21], [22],[22], [22], [23], [24]] or unsupervised [[25], [26], [27]], limiting the scalability and accessibility to the intervention. Individuals recovering from mTBI encounter multifaceted barriers to physical activity participation that extend beyond the injury itself. Traditional in-person exercise programs may be particularly challenging for this population due to symptom exacerbation from travel, sensory sensitivities in gym environments, cognitive demands of navigating unfamiliar spaces, and executive function capacity required to coordinate going to an in-person exercise intervention. Virtual exercise interventions addresses these limitations, having demonstrated effectiveness and feasibility across diverse populations facing similar accessibility challenges, including individuals in rural communities with limited healthcare access and cancer survivors managing treatment-related fatigue and mobility restrictions [[28], [29], [30], [31], [32], [33], [34], [35]].

Here, we developed and will test an individualized, supervised, virtual, symptom-thresholded aerobic exercise intervention that directly addresses several barriers to participation by providing personalized, home-based exercise delivery that adapts to participants' fluctuating symptoms while accommodating for their unique schedules and environments. This approach eliminates transportation barriers while allowing real-time modification of exercise intensity based on symptom presentation. This protocol leverages previously established exercise tolerance tests, such as the Buffalo Concussion Treadmill Test [36], which have demonstrated validity in sport related concussion and in community dwelling individuals with mTBI [37,38]. While these tests have traditionally guided return-to-play for athletes, our approach applies this methodology specifically to non-sport related concussion and guides real-time engagement in physical activity interventions rather than serving as only a single use determinant for unrestrained and higher-risk activity. This individualized approach minimizes the risk of symptom exacerbation while fostering self-efficacy and confidence in physical activity engagement to promote lifelong engagement, particularly important for exercise-naïve individuals who may harbor concerns about their physical capabilities following brain injury.

The complex pathophysiology of TBI involves both immediate damage to the brain and prolonged secondary injury cascades, including neuroinflammation, oxidative stress, and disrupted cerebral metabolism [39,40]. These processes alter brain structure and function [[41], [42], [43], [44], [45], [46], [47], [48], [49], [50]], impeding natural recovery mechanisms, and create an urgent need for interventions that can counteract these detrimental effects. Emerging evidence suggests that physical activity and exercise may serve as a therapeutic tool by targeting multiple aspects of TBI pathophysiology simultaneously. In preclinical models, aerobic exercise demonstrates neuroprotective and neurorestorative properties, mitigating neuroinflammation, enhancing neurogenesis, and promoting synaptic plasticity [[51], [52], [53], [54], [55], [56], [57]]. Despite compelling animal model research on the neural responses to exercise following TBI, current clinical work primarily focuses on symptom improvement without addressing underlying neural damage [[58], [59], [60]]. Promoting recovery mechanisms in people recovering from TBI could directly address the secondary injury mechanisms that perpetuate persistent cognitive dysfunction following mTBI. By employing PA, it is our objective to bolster endogenous recovery mechanisms and directly counteract secondary injury mechanisms that perpetuate persistent cognitive dysfunction following mTBI.

Multimodal neuroimaging is also crucial for improving the understanding of exercise's mechanistic effect on TBI as it can capture the neural responses demonstrated in prior preclinical models [61]. Comprehensive imaging is particularly valuable given that exercise appears to affect multiple neural substrates simultaneously, suggesting a common underlying mechanism rather than isolated effects [49,62,63]. By using complementary imaging modalities, we can develop models of exercise induced recovery in mTBI, identify biomarkers for personalized exercise interventions, and bridge the translational gap between promising animal findings and human clinical applications. This would allow for TBI management to transform from a symptom management approaches to treatments that directly address the underlying neural injury while simultaneously treating symptoms in combination.

In 2022, The Lancet published key messages and recommendations for the future of TBI research [1]. Specifically, Table 1, section 3 called for improved access to rehabilitation services and develop evidence-based treatments for long-term problems, including fatigue, and cognitive and behavioral change [1]. This proposed study has the potential to improve accessibility to rehabilitation services and significantly contribute to the development of evidence-based treatment for cognitive problems. Thus, it is well posed to have a sizable impact on substantial gaps in the current literature, providing the basis for clinical care and future recommendations.

**Table 1:** Inclusion-Exclusion Criteria for study participants.

Inclusion	Exclusion
Clinical diagnosis of mTBI (<1 year)	Diagnosis of moderate/severe TBI; history of mTBI > 1year
18–55 years of age	Presentation of skull breach or subdural hematoma
People of all gender identities, ethnicities/races, and socioeconomic status	Prior diagnosis of cognitive or physical disability
Ability to provide informed consent	Clinical diagnosis of other neurological or neuropsychiatric disorder
Safe to exercise (based answering “no” to all questions on the Physical Activity Readiness Questionnaire (PAR-Q))	Treatment for congestive heart failure, angina, uncontrolled arrhythmia, or other cardiovascular events
Normal or corrected to normal vision	History of myocardial infarction, coronary artery bypass grafting, angioplasty, or other cardiac condition in the last year
Able to speak, read, and write English	Regular use of assisted walking device
Ambulatory without pain or assistance of walking device	Not fluent in English
No diagnosis of comorbid neurological condition	Not medically cleared for exercise
MRI compatible (no metal or contraindications)	Not MRI compatible
No history of brain bleeds	

### 1.1 Summary of aims

The primary aim is to establish the feasibility of delivering a virtual exercise intervention in community-dwelling individuals with mTBI, including assessment of recruitment rates, adherence, retention, and safety parameters. The secondary aim establishes precision around the mean and variance [64] of a symptom-guided aerobic exercise intervention to change cognitive function and metrics of brain structure and function following recent (within 1-year of injury) mTBI. Two exploratory aims will investigate: (1) the precision around the mean and variance of aerobic exercise's effect on cognitive function; and (2) the precision around the mean and variance of aerobic exercise's effect on neuroimaging measures of brain structure, function, connectivity, and perfusion. A final exploratory aim is to measure changes in objectively measured daily physical activity and sedentary behaviors measured three times across the intervention period and their associations with neurological and cognitive outcomes.

## 2 Methods

### 2.1 Study design

**Fig. 1:**
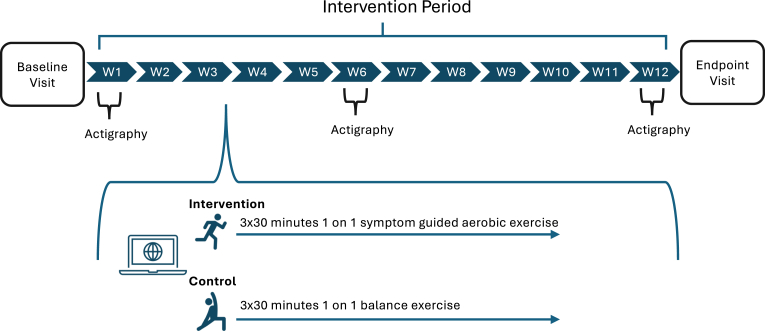
Overall Study Design. Baseline is followed by 12-week intervention period with actigraphy at Week 1, Week 6, and Week 12. After completion, an endpoint visit is conducted.

The current study is a stratified block two-arm pilot randomized controlled trial design with multiple complementary objectives. After reading and signing informed consent, recruited participants are randomized into either an aerobic exercise (intervention) or balance exercise group (control) with a ratio of 1:1 (Fig. 1). All participants are informed about the randomization but blinded to their group assignment, meaning they are unaware which group is intended to be intervention. Participants are informed that the study aims to examine whether a 12-week exercise program promotes brain health and cognition after an mTBI. However, it is not possible to blind experimenters, as they lead the intervention sessions and give instructions during the assessments. The randomization scheme is prepared by the Tufts Clinical and Translational Science Institute Group using a permuted block method with random blocks of 4 and 6. Randomized block design is used to increase experimental precision and reduce the impact of unwanted variability (nuisance factors) by grouping similar subjects or experimental units into blocks before randomly assigning treatments within each block. This trial is registered on clinicaltrials.gov under identifier NCT06494592. This study received ethical approval from Northeastern University Institutional Review Board (IRB).

### 2.2 Eligibility

The eligibility criteria are designed to recruit individuals who have a clinical diagnosis of mTBI within the last previous year and can safely engage in aerobic exercise (Table 1).

### 2.3 Recruitment

We will recruit participants until a final sample of 24 has completed all 12 weeks. Adults aged 18–55 years who have sustained a clinically diagnosed mTBI, confirmed by a neurologist (AS), within the past year are eligible to participate. Complete inclusion and exclusion criteria are detailed in Table 1. To ensure representative findings, we will actively recruit a diverse participant population with balanced representation across race, sex, and socioeconomic status. Recruitment strategies include multi-modal advertising approaches, including promotional flyers distributed throughout healthcare facilities, community centers, and university campuses in the greater Boston metropolitan area. Additionally, targeted online recruitment will be conducted through social media platforms (e.g., Facebook) and community-based websites (e.g., Craigslist) to maximize reach across diverse demographic groups. We will also establish partnerships with local healthcare providers, rehabilitation centers, and concussion clinics to facilitate referrals of eligible participants.

### 2.4 Screening

Questions about medical history, safety to exercise [65] and MRI Safety are asked in the initial phone screen by research staff. Participants are required to be diagnosed with a mTBI within the last one year, cleared to exercise, and be safe for an MRI scan before enrolling. If participants do not have a clinical diagnosis, our clinician (AS) will evaluate them for mTBI via phone interview and OSU TBI Questionnaire.

### 2.5 Randomization

Participants are randomized after the baseline session is complete using a block randomization in block sizes of four and six with equal allocation to each group. Randomization were done in REDcap.

### 2.6 Cognitive testing

The cognitive tests were chosen to provide measures of domains with known deficits after a mTBI and which are commonly used in clinical practice, and were administered at baseline and endpoint. Hopkins Verbal Learning Test (HVLT) [66] provides measures of memory, including immediate and delayed recall. Letter [67] and category fluency [68] provides measures of language. The Trail Making Test (TMT) [69] A and B provides measures of processing speed and executive functioning.

### 2.7 Symptom threshold and cardiorespiratory fitness testing

**Fig. 2:**
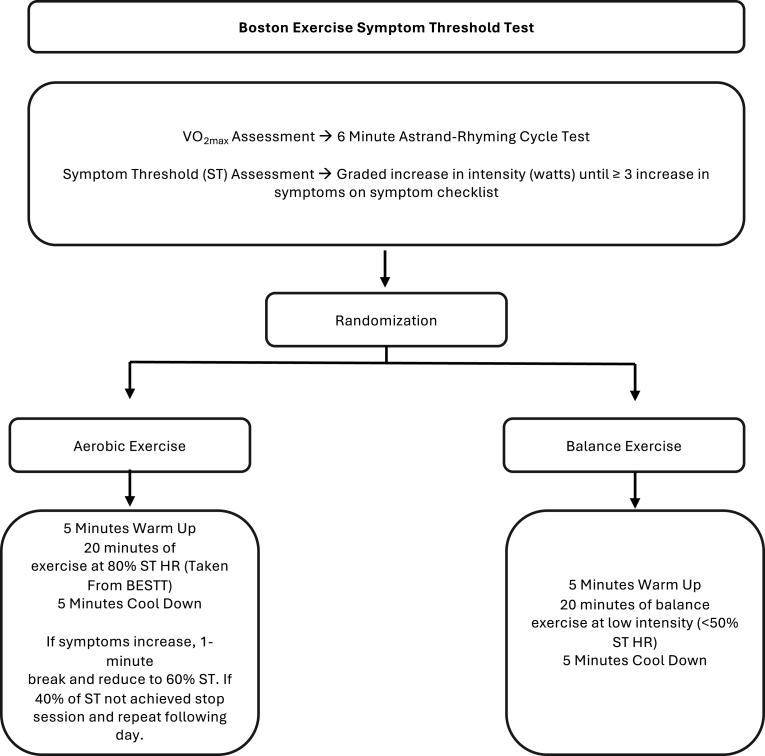
Boston Exercise Symptom Threshold Test Timeline and decision tree.

We developed an in person hybrid symptom threshold test (similar to the Buffalo concussion treadmill test and others [36,70]) to objectively determine the exercise intensity (and heart rate) at which concussion-associated symptom severity increases beyond baseline levels (defined as an increase of ≥3 units above baseline), and combined sub-maximal cardiorespiratory fitness estimation of VO_2_max (based on the Astrand-Rhyming 6-min protocol [71]) (Fig. 2). Together, these assessments will determine the appropriate intensity for aerobic exercise sessions in the intervention group and provide a pre-post measure of CRF. The complete assessment (administered at baseline and post-intervention) consists of three sequential phases conducted on a cycle ergometer: warm-up, Astrand-Rhyming test [72], and symptom threshold test. Baseline vital signs (heart rate, blood pressure) are recorded during seated rest. Participants complete a graded concussion symptom checklist to establish baseline symptom ratings. Heart rate (Polar H7 chest strap, Polar, Kempele, Finland), ratings of perceived exertion (RPE; Borg scale), and concussion symptoms are assessed every 2 min during all phases using the CIF. During Phase 1 (Warm-up) participants cycle at self-selected resistance and pace for 7 min. Then, Phase II (Astrand Rhyming Test) begins. Participants cycle at 50 revolutions per minute (RPM) at a predetermined workload (calculated in watts based on participant weight) for 6 min. The cycle ergometer automatically calculates workload in watts for subsequent VO_2_max estimation using the Astrand-Rhyming nomogram. The average heart rate during the fifth minute is recorded. The test phase concludes when steady-state heart rate is achieved (defined as heart rate variations ≤10 beats per minute). The steady-state heart rate and workload are used to estimate VO_2_max via nomogram extrapolation. Upon completion of the VO_2_max estimation, participants are instructed to increase their pedal cadence to 60 RPM. Resistance increases every 30 s until one of the following termination criteria is met: increase of ≥3 symptoms above baseline levels, 85 % of age-predicted maximum heart rate or RPE of 18 on the Borg scale, any relative or absolute contraindication to exercise testing. The heart rate at symptom threshold is recorded and used to prescribe aerobic exercise intensity for intervention sessions.

### 2.8 Balance test

The Balance Error Scoring System (BESS) [73] will be used to evaluate postural stability and administered by trained research staff. The BESS protocol consists of three stance positions performed on two different surfaces (firm and foam), yielding six total testing conditions. The three stance positions include double-limb stance with feet together in a narrow base of support, single-limb stance on the non-dominant leg only, and tandem stance with the non-dominant foot positioned behind the dominant foot in heel-to-toe alignment. Each stance is performed on both a firm surface (standard flat ground) and a foam surface (medium-density foam pad). Each of the six conditions is performed for 20 s with eyes closed while a trained evaluator observes and scores each trial by counting balance errors. Balance errors are defined as opening eyes, lifting hands off hips, stepping, stumbling, or falling, lifting the forefoot or heel off the ground, or hip abduction or flexion greater than 30°.

### 2.9 Brain magnetic resonance imaging

To assess the impact of exercise on brain morphology, function, connectivity, white matter microstructure, and cerebral blood flow, a magnetic resonance imaging (MRI) is completed at baseline and endpoint. A Siemens Prisma 3T scanner with a 64-channel head coil will be used to complete MRI scans. Structural imaging consisted of high-resolution three-dimensional (3D) anatomical sequences including a Magnetization-Prepared Rapid Acquisition with Gradient Echo (MPRAGE) T1-weighted sequence with navigator correction (sagittal orientation, 0.8 mm isotropic resolution, TE/TI/TR = 1.81–7.18/1000/2500 ms, field of view (FOV) 256 mm × 240 mm, 208 slices, GRAPPA acceleration factor = 2). An anatomical scout sequence was also acquired for positioning (sagittal orientation, 1.6 mm isotropic resolution, TE/TR = 1.37/3.15 ms, FOV 260 mm, 128 slices, GRAPPA acceleration factor = 3). Diffusion-weighted imaging (DWI) utilized a high-resolution multi-shell sequence with anterior-posterior (AP) and posterior-anterior (PA) phase encoding directions for distortion correction (1.5 mm isotropic resolution, TE/TR = 89.20/3230 ms, multiband acceleration factor = 4, FOV 210 mm, 92 slices, b-values of 0 and 3000 s/mm^2^, 98 gradient directions, monopolar diffusion scheme). Functional MRI included a resting-state Echo Planar Imaging (EPI) sequence (2.0 mm isotropic resolution, TE/TR = 37/800 ms, multiband acceleration factor = 8, FOV 208 mm, 72 slices, 600 measurements, PA phase encoding direction). Field mapping sequences for distortion correction were acquired with matching geometry (2.0 mm isotropic resolution, TE/TR = 66/8000 ms, FOV 208 mm, 72 slices) in both AP and PA phase encoding directions. Arterial spin labeling (ASL) perfusion imaging employed a pseudo-continuous ASL (pCASL) sequence with multiple post-labeling delays (2.5 × 2.5 × 3.0 mm resolution, TE/TR = 36.48/4100 ms, transversal orientation, FOV 240 mm, 42 slices, flip angle = 120°, fat saturation, post-labeling delays = 500, 1000, 1500, 2000, 2500 ms, 13 measurements total including calibration scans).Data will undergo quality control assessments for sequency accuracy (naming standards, correct number of volumes, correct sequence parameters). Then, data are converted to NIfTI format and structured per the Brain Imaging Data Structure (BIDS). Structural and functional data will then be preprocessed using fmriprep [74]. DWI data will be preprocessed using MRtrix3 [75]. ASL data will be preprocessed using ExploreASL [76]. A visual inspection of the data is done for quality control purposes to ensure preprocessing steps were completed without errors.

### 2.10 Electroencephalography

An EMOTIV FLEX 2 Saline - 32 Channel Wireless EEG Head Cap System was used to assess the effects of exercise on changes in brain function. The electrode montage is based on the 10–20 international system [77,78]. 10 min of resting state electroencephalography (rs-EEG) will be conducted where participants will be instructed to focus on a fixation point in the middle of the screen for 5 min during the eyes open condition and will be instructed to have their eyes closed for 5 min during the eyes closed condition. Each participant will be positioned 41 cm from the screen to achieve homogeneity in rs-EEG collection. Data will be pre-processed using a custom MATLAB script. Data will undergo filtering, re-referencing to the average of all electrode sites, bad channel rejection, independent component analysis (ICA) deconstruction, eye-correction, ICA component removal, segment rejection, and interpolation. Post-processing will include a spectral power analysis using fast Fourier time-to-frequency domain transformation. Boundaries for the canonical frequency bands are as follows: delta (1–4hz), theta (4–8hz), alpha (8–13hz), beta (14–30hz), and gamma (31–45hz) [79].Power spectral metrics will also be input into a custom Python script including fitting oscillations one over f (FOOOF) [80]. This will be done to obtain aperiodic metrics of brain function.

### 2.11 Psychosocial assessments

A battery of questionnaires are administered at baseline and endpoint including quality of life and lifestyle behaviors questionnaires (see Table 2). Additionally, pre-injury behaviors are of interest to see how engagement in healthy lifestyle behaviors pre-injury may contribute to recovery from mTBI. As such, we will administer a modified International Physical Activity Questionnaire (IPAQ), asking specifically about pre-injury physical activity engagement.

**Table 2:** Study assessments and timeline.

	Phone Screen	Baseline (in person)	Randomization	Week 6	Week 12	Endpoint (in person)
Prescreening Questionnaire	X					
The Physical Activity Readiness Questionnaire (PAR-Q)	X					
Informed Consent		X				
Demographics		X				
The Ohio State University Traumatic Brain Injury Identification Method (OSU TBI-ID) Questionnaire		X				
Hopkins Verbal Learning Test (HVLT)		X				X
Patient-Reported Outcomes Measurement Information System (PROMIS)		X				X
Menstrual History Questionnaire [81]		X				
International Physical Activity Questionnaire (IPAQ)		X				
Pittsburg Sleep Quality Index (PSQI)		X				X
Verbal Fluency		X				X
Mediterranean Diet Adherence		X				
Trail Making Test (TMT) A and B		X				X
Magnetic Resonance Imaging		X				X
Electroencephalography		X				X
Balance Error Scoring System (BESS)		X				X
Cardiorespiratory Fitness Test		X				X
Actigraphy		X		X	X	

### 2.12 Physical activity monitoring

Physical activity, sedentary time, and sleep metrics will be measured using an GT9X Link accelerometer (ActiGraph LLC, Pensacola, FL). An accelerometer is worn on the non-dominant wrist to improve compliance and allow for assessment of sleep characteristics. Time spent in physical activity, as well as time spent in light, moderate, and vigorous intensities are measured. Participants are also asked to complete a sleep log and record times when they remove and the device during each day (e.g., before and after taking shower). Actigraphs will be uploaded using ActiLife and preprocessed using R package *ggir* [82].

### 2.13 Study assessments

See Table 2 for complete schedule of all study assessments.

### 2.14 Intervention

All participants will engage in staff-led virtual exercise interventions three times a week for 30-min (5-min warm up, 20-min of aerobic/balance exercises, 5-min cooldown) for 12 weeks, making a total of 36 intervention sessions. Three sets of five exercises with modifications per each exercise have been developed for each intervention group (balance and aerobic) to help maintain engagement and allow intensity of the exercise to progress (see Table 3). Participants are seen individually for intervention sessions to ensure the research team can individualize exercise guidance and for safety monitoring All virtual sessions will be administered by two trained research staff members, one who leads the exercise session and one safety observer, monitoring safety. Heart rate (HR) will be monitored and recorded by hyperate4health (https://hyperate4health.netlify.app/), a proprietary open access platform and participants will be asked to rate their symptoms and exertion every 5 min. By collecting symptoms, HR using a Polar chest strap, and RPE every 5 min, we are directly targeting key limitations in other aerobic exercises studies done in TBI populations [18]. Aerobic sessions have a goal of reaching 20 min of aerobic exercise performed at 80 % of HR at symptom threshold. If this intensity increases symptoms by +3 on the symptom checklist, a 1-min break is taken, and the goal is reduced to 60 % of symptom threshold HR. If this intensity still exacerbates symptoms, another 1-min break is taken, and exercises will be done with a goal of 40 % of symptom threshold HR. Virtual aerobic exercises have been developed by an American College of Sport's Medicine certified Exercise Physiologist (JC) with experience delivering virtual aerobic interventions in older adults [83]. All participants are given a Polar HR strap and instructions on how to connect to HypeRate via Bluetooth to monitor their HR. Participants in the balance group are sent home with a foam pad to engage in non-aerobic physical activity like balance exercises. Each exercise is progressive in nature and includes modifications. All balance exercises were developed by a physical therapist (JM) and commonly used in clinical practice.

**Table 3:** Intervention description.

Exercise description	Modification
Aerobic Version A	
Standing Twists w/Arm push	Normal, high arm, high arm with speed
Half Jacks	One arm, both arms, with jump
Imaginary Ball Slams	Normal, on toes, touch ground
Lateral Shuffles	Normal, Arms overhead, arms overhead with speed
Imaginary Kettlebell swings	Normal, Side steps, forward steps
Aerobic Version B	
Large step to back shuffle	Step, Leap, leap with arms overhead
Skaters	Normal. Touch ground, ground to overhead
Punching knees bents	Normal, jab cross/hook, jab cross/knee
Skiers	Small steps, large steps, high arms with steps
Elbow to knee	Elbow only, elbow to knee, add hop
Aerobic Version C	
Toe Taps	Hands on hips, high arms, high arms with speed
Standing dead bugs	Opposite arm/leg, overhead arm/opposite leg, both arms overhead
One leg diagonal step	Slight bent knee, arms to side, arms overhead
V-Steps	Normal, arms overhead with step, arms overhead always
Step overs	Normal, with arms pulling, arms overhead
Balance Version A	
Seated reach outside base of support	Normal, Foam Pad
Seated Wood Choppers	Normal, Foam Pad
Standing reach outside base of support	Normal, Foam Pad
Standing eyes closed	Normal, foam pad, narrow base of support
Standing eyes open on foam pad	Normal, narrow base of support, single leg
Balance Version B	
Seated marches	Normal, Foam Pad
Seated Crossbody Oblique Dips	Normal, Foam Pad
Standing Marches	Normal, Foam Pad
Tandem Stance	Eyes open, eyes closed
Clocks	Normal, foam pad
Balance Version C	
Seated cat cow	Normal, Foam Pad
Rotating knee taps	Normal, Foam Pad
Calf raises	Normal, Foam Pad
Drinking birds	Normal, Foam Pad
Tandem foot placement	Eyes open, eyes closed

### 2.15 Monitoring adverse events

All adverse events are monitored and managed throughout the trial. All events will be reported in future manuscripts. The role of the safety observer is to monitor the wellbeing of each participant and respond to falls, signs of distress, or other safety concerns. Before a session begins, the safety observer will: 1) have the spreadsheet open/available with names, addresses, and contact information of the participant and their local emergency contact, 2) have phone readily available to call participants, 3) confirm participants are in their address previously collected, 4) instruct participants that they must announce to the study staff if they are going to leave the screen (to get water, to sit down or to take a bathroom break), 5) check for room safety, 6) ensure chair must not have wheels, 7) ensure participant is either fully on or fully off a rug, and 8) ensure no objects in way of exercise space. During the session, safety observer will: 1) observe for flushed face, increased perfusion, pale features and 2) Monitor HR and record data on RedCAP.

### 2.16 Data management and quality control

All research data are de-identified and only the research team has access to identifiable information that is stored in a secure location. Data are stored electronically on password protected servers behind university-protected firewalls. Data are stored in two ways: (1) MRI data and physical activity monitoring data are extracted from software packages and uploaded directly to a secure storage environment at a high-performance computing facility (Northeastern University Research Computing Cluster); (2) All cognitive and psychosocial assessments are collected in REDcap [84].

### 2.17 Post study report and survey

**Fig. 3:**
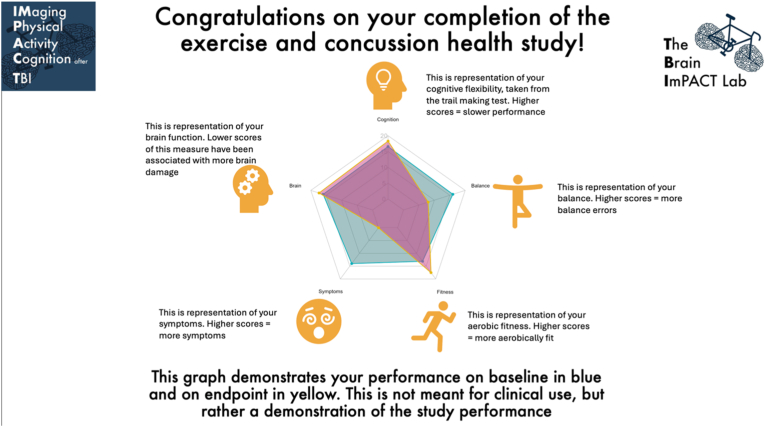
Example of the post study report given to each participant upon completion of the study.

After participants complete the study, they receive a post study report (Fig. 3) and feedback survey to fill out. The post-study report allows participants to receive feedback on their progress throughout the study. The cognition on the survey is TMT-B completion time. The balance is BESS total score. Fitness is CRF determined from Astrand-Rhyming test. Symptoms are the CIF total symptom score at baseline before the Astrand-Rhyming test. Brain function is alpha peak frequency from EEG. The feedback survey allows for the research team to receive feedback and make improvements to the study and future studies, as well as collecting data on reasons for low adherence and dropouts.

### 2.18 Analysis plan

The goal of the pilot study is to complete 24 participants (12 in each group). Data will be checked for completeness and correctness using frequency distributions. Group differences at baseline will be examined for demographic factors, baseline symptoms, baseline estimated CRF, baseline balance, and baseline cognition to detect potential confounding factors. For the primary feasibility aim, we will report dropouts, adherence rates, retention rates, adverse events, and compliance. Statistical models to test for differences in outcomes over time between groups will be used but inferences will not be made based on p-values given the sample size was chosen based on feasibility and precision and variance around the mean difference. Therefore, all models will be reported as effect sizes with standard error of the means and 95 % confidence intervals around the mean.

## 3 Discussion

Post-injury aerobic exercise interventions to improve cognitive dysfunction following mTBI have the potential to have a considerable impact on recovery post mTBI. We have designed a 12-week pilot randomized controlled trial to examine the efficacy of a virtual, symptom threshold aerobic exercise intervention on cognitive recovery in mTBI populations. While we are not powered to detect true differences in cognitive and brain outcomes between groups over time, we will explore these outcomes to inform larger studies.

Several limitations should be acknowledged in interpreting the findings of this pilot study. The modest sample size (n = 24) inherently limits statistical power and may be insufficient to detect clinically meaningful effects for secondary and exploratory neuroimaging outcomes, though it is appropriate for establishing feasibility and generating preliminary effect size estimates (precision and variability around the mean) for future adequately powered trials. Geographic recruitment from the greater Boston area and English-language requirements may limit the generalizability of findings to more diverse populations and geographic regions. The virtual delivery format, while offering advantages in accessibility and scalability, introduces unique methodological challenges. Participants may experience technological barriers, home environment distractions, or suboptimal exercise execution due to limited real-time supervision. These factors could potentially reduce intervention fidelity and exercise intensity compared to traditional in-person supervised training. Additionally, maintaining participant motivation and adherence over the 12-week intervention period may prove challenging in a home-based setting without the social support and accountability of group-based programs. The heterogeneous nature of the mTBI population presents additional considerations. The broad enrollment window (up to one-year post-injury) captures individuals at varying stages of natural recovery, potentially introducing variability in baseline function and treatment responsiveness. Furthermore, the subjective nature of symptom reporting used for exercise prescription may introduce inconsistencies, as individuals may vary in their symptom awareness, reporting accuracy, or willingness to report symptom exacerbation. Finally, while the balance control group provides an active comparator to control for attention and social contact effects, these exercises may confer some cognitive benefits, potentially attenuating between-group differences. Despite these limitations, this pilot study will provide crucial feasibility data and preliminary efficacy estimates to inform the design of future definitive trials.

In summary, this 12-week pilot randomized controlled trial will evaluate the feasibility and preliminary efficacy of a symptom-guided virtual aerobic exercise intervention for improving cognitive function following mTBI. This study addresses a critical gap in evidence-based treatments for cognitive recovery post-mTBI by combining innovative virtual delivery with individualized exercise prescription based on objective symptom thresholds. The findings will establish the feasibility of recruiting, randomizing, and retaining participants in a virtual exercise intervention while providing preliminary evidence regarding the potential cognitive benefits of aerobic exercise compared to an active balance control condition. Additionally, the comprehensive assessment battery will yield valuable insights into the neurobiological mechanisms underlying exercise-induced cognitive improvements through advanced neuroimaging measures of brain structure, function, connectivity, and perfusion. The results will inform the design and implementation of future large-scale randomized controlled trials by providing essential feasibility metrics, effect size estimates, and methodological refinements necessary for definitive efficacy testing. Ultimately, this research may contribute to the development of accessible, evidence-based interventions that can be readily implemented in clinical practice to support cognitive recovery in the substantial population of individuals affected by mTBI.

## CRediT authorship contribution statement

**Emma M. Tinney:** Writing – original draft, Visualization, Validation, Supervision, Project administration, Methodology, Investigation, Formal analysis, Data curation, Conceptualization. **Mark C. Nwakamma:** Writing – review & editing, Project administration, Methodology, Investigation, Formal analysis, Data curation, Conceptualization. **Goretti España-Irla:** Writing – review & editing, Data curation. **Madeleine Perko:** Writing – review & editing, Data curation. **Ryan Luke Sodemann:** Writing – review & editing, Data curation. **Jacqueline Caefer:** Writing – review & editing, Methodology, Data curation. **Julia Manczurowsky:** Writing – review & editing, Methodology. **Charles H. Hillman:** Writing – review & editing, Supervision, Conceptualization. **Alexandra Stillman:** Writing – review & editing, Methodology. **Timothy P. Morris:** Writing – review & editing, Visualization, Validation, Supervision, Software, Resources, Project administration, Methodology, Investigation, Funding acquisition, Formal analysis, Conceptualization.

## Declaration of competing interest

The authors declare that they have no known competing financial interests or personal relationships that could have appeared to influence the work reported in this paper.

## Data Availability

No data was used for the research described in the article.
